# Combination immunotherapy in a patient with hemodialysis therapy and metachronous bilateral clear cell renal cell carcinoma: Case report and literature review

**DOI:** 10.1016/j.eucr.2022.102242

**Published:** 2022-09-24

**Authors:** Alejandro Pineda Isaza, Alvaro Osorio Franco, Lisceth Paola Quintero González, Marcela Vallejo Fajardo

**Affiliations:** a Fellow of Hematology and Clinical Oncology, Health Sciences Faculty, Icesi University, Cali, Colombia; b Hematologist and Clinical Oncologist, Department of Hematoncology, Department of Internal Medicine, Fundación Valle del Lili, Cali, Colombia; c Internal Medicine Resident. Health Sciences Faculty, Icesi University, Cali, Colombia; d Clinical Oncologist. Department of Hematoncology, Department of Internal Medicine, Fundación Valle del Lili, Cali, Colombia

**Keywords:** Renal cell carcinoma, Immunotherapy, Hemodialysis

## Abstract

Combination immunotherapy is a treatment strategy in patients with renal cell carcinoma that has proved to be effective in phase III randomized clinical trials. These studies do not include patients with end stage kidney disease on hemodialysis. We discuss this case about a patient with metachronous bilateral clear cell renal cell carcinoma, managed with bilateral nephrectomy and ulterior requirement of hemodialysis, with lung and intestinal progression, managed with combination immunotherapy, with a partial response and absence of adverse effects related to treatment.

## 1 Introduction

Renal cell carcinoma (RCC) comprises a heterogeneous group of renal tubular epithelial cells cancers and represents almost 4% of malign tumors in adults. One third of RCC patients who are taken to local surgical resection, have tumor relapse with appearance of distant metastases.^1^

Since 2005, tyrosine kinase inhibitors, mTOR inhibitors and immune checkpoint inhibitors play a central role in the treatment of this disease; randomized clinical trials have evidenced significant increase in global and progression-free survival.^1^ We herein report a case of metachronous bilateral clear cell renal cell carcinoma in a patient with hemodialysis therapy treated with combination immunotherapy.

## 2 Case presentation

A 61-year-old male patient with a history of grade 2-pT1aNxM0 clear cell renal cell carcinoma (RCC) of 2 × 3 cm in the middle lobe of his right kidney diagnosed in 2014, treated with right radical nephrectomy at that time, and followed in regular consultations with computed tomography (CT).

In 2018, a CT identified a nodular image in the upper lobe of the left kidney; hence a percutaneous biopsy was performed and the pathology was consistent with grade 1 clear cell RCC. Surveillance was not considered due to concerns of the patient. Again, the patient required a radical left nephrectomy and his oncologic disease was classified grade 2 pT1aNxM0. Since then, he required hemodialysis therapy and regular oncology assessments in another health center in the city, his functionality was preserved, ECOG 0.

In July 2020, he was found hypotensive before his hemodialysis therapy, he complained with dizziness, melena, asthenia and tiredness during the past three weeks; therefore he was remitted to our hospital emergency department. At his admission, he was tachycardic, hypotensive and pale, his initial tests showed severe microcytic and hypochromic anemia, thus requiring 2 red cells units transfusion and fluid support therapy. An upper endoscopy with biopsy was performed and it evidenced a protruded, irregular, ulcerated and friable 10 mm lesion at the posterior wall of the duodenal angle, it occluded 50% of the luminal area. The pathology revealed extensively ulcerated duodenal mucosa, infiltrated by malign epithelial intermediate tumor cells with clear cytoplasm, and intermediate, irregular and hyperchromatic nuclei, which were arranged on nests and cords. Immunohistochemical staining was positive for CKAE1/AE3, CD10 y PAX8, and negative for CK7, CK20 and RCC, which was consistent with metastatic RCC. Thorax and abdomen CT documented multiple metastatic lesions at the right liver lobe, as in both lungs and mediastinum.

The melenic stools persisted and hemostatic endoscopic measures were insufficient, therefore an embolization was performed, and the bleeding ceased.

**Fig. 1:**
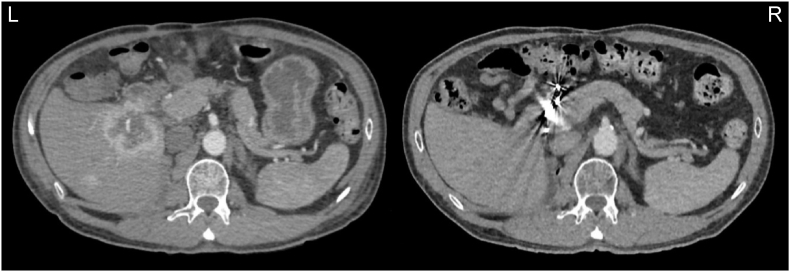
Abdominal CT before combination therapy with nivolumab plus ipilimumab (left) and after 6 months (right), showing significant reduction of liver metastases.

Combination immunotherapy was selected according to the bleeding risk and adverse effects, thus pembrolizumab plus axitinib were excluded, and nivolumab + ipilimumab were preferred. The cycles were administered 3 hours after the end of the hemodialysis session. He achieved four full dose cycles and single nivolumab therapy was maintained. A new CT was performed after 14 months (Fig. 1), the lung and liver lesions disappeared, and there was a 16 × 17 mm duodenum nodular lesion, another metastatic lesion were not found.

## 3 Discussion

The phase III randomized clinical trial Checkmate 214 compared combination of two immune checkpoint inhibitors: PD-1 inhibitor nivolumab plus CTLA-4 inhibitor ipilimumab, against tyrosine kinase inhibitor sunitinib in naïve patients with metastatic RCC. A total of 1096 patients were assigned to receive nivolumab (3 mg per kilogram of body weight) plus ipilimumab (1 mg per kilogram) intravenously every 3 weeks for four doses followed by nivolumab (3 mg per kilogram) every 2 weeks, or sunitinib (50 mg) orally once daily for 4 weeks (6-week cycle). At a median follow-up of 25.2 months in intermediate- and poor-risk patients, the 18-month overall survival rate was 75% (95% confidence interval [CI], 70 to 78) with nivolumab plus ipilimumab and 60% (95% CI, 55 to 65) with sunitinib; the median overall survival was not reached with nivolumab plus ipilimumab versus 26.0 months with sunitinib (hazard ratio for death, 0.63; P < 0.001). The objective response rate was 42% versus 27% (P < 0.001), and the complete response rate was 9% versus 1%. The median progression-free survival was 11.6 months and 8.4 months, respectively (hazard ratio for disease progression or death, 0.82; P = 0.03, not significant per the prespecified 0.009 threshold). The trial protocol excluded patients with glomerular filtration rate under 40 ml/min/1.73 m2 according to Cockroft & Gault formula.^2^

A clinically significant impact on pharmacokinetics of nivolumab and ipilimumab has not been observed in patients with end stage renal disease.^3^ This subgroup of patients is underrepresented or excluded in different clinical trials; consequently safety and efficacy data of immune checkpoint inhibitors in hemodialysis patients are insufficient.^4^

Combination immunotherapy lacks evidence in RCC and hemodialysis cases, there are only case reports and cases series. Kobayashi et al.^5^ reported a case of a 77year-old patient with end stage kidney disease associated to hyperuricemia, in hemodialysis three times a week, with clear cell RCC and right radical nephrectomy, with lung metastatic disease after four years. He received nivolumab 240 mg plus imilimumab 1 mg/kg intravenously every three weeks for four doses, followed by nivolumab 240 mg every two weeks. Follow-up CT showed stable disease after 8 months and adverse events were minimal.

## 4 Conclusions

RCC is a relevant disease, whose treatment has changed over the years, and immune checkpoint inhibitors have a central role, as monotherapy or combination. Plenty evidence is lacking about the real efficacy and safety of combination immunotherapy in patients with RCC and hemodialysis requirement, only supported by case reports. Further clinical trials are required to answer this clinical question.

## Ethical issues

The authors state that this study is not considered hazardous research according to international rand national regulations. Data collection was obtained from clinical records.

## Financing

The authors financed the article.

## Declaration of competing interest

The authors disclaim interests’ conflict related to this article.
